# PARP1 Might Substitute HSF1 to Reactivate Latent HIV-1 by Binding to Heat Shock Element

**DOI:** 10.3390/cells11152331

**Published:** 2022-07-29

**Authors:** Xinfeng Xu, Yingtong Lin, Xiaoyun Zeng, Chan Yang, Siqin Duan, Liqiong Ding, Wanzhen Lu, Jian Lin, Xiaoyan Pan, Xiancai Ma, Shuwen Liu

**Affiliations:** 1 Guangdong Provincial Key Laboratory of New Drug Screening, Guangzhou Key Laboratory of Drug Research for Emerging Virus Prevention and Treatment, School of Pharmaceutical Sciences, Southern Medical University, Guangzhou 510515, China; xxf7665@smu.edu.cn (X.X.); linyingt27@163.com (Y.L.); xiaoyunzeng106@163.com (X.Z.); virus6522@smu.edu.cn (C.Y.); dsq2013@i.smu.edu.cn (S.D.); dinglq2021@163.com (L.D.); wanzhenlu0111@gmail.com (W.L.); linjian201111@163.com (J.L.); 2 Institute of Human Virology, Department of Pathogen Biology and Biosecurity, Key Laboratory of Tropical Disease Control of Ministry of Education, Zhongshan School of Medicine, Sun Yat-sen University, Guangzhou 510080, China; 3 Department of Pharmacy, The Seventh Affiliated Hospital of Sun Yat-sen University, Shenzhen 518107, China; 4 School of Pharmaceutical Sciences, Hubei University of Science and Technology, Xianning 437100, China; 5 Center for Biosafety Mega-Science, State Key Laboratory of Virology, Wuhan Institute of Virology, Chinese Academy of Sciences, Wuhan 430071, China; panxy@wh.iov.cn; 6 Guangzhou Laboratory, Guangzhou International Bio-Island, Guangzhou 510005, China

**Keywords:** HIV-1 latency, latency reversing agents, heat shock factor 1, heat shock element, functional cure, PARP1

## Abstract

At present, the barrier to HIV-1 functional cure is the persistence of HIV-1 reservoirs. The “shock (reversing latency) and kill (antiretroviral therapy)” strategy sheds light on reducing or eliminating the latent reservoir of HIV-1. However, the current limits of latency-reversing agents (LRAs) are their toxicity or side effects, which limit their practicability pharmacologically and immunologically. Our previous research found that HSF1 is a key transcriptional regulatory factor in the reversion of HIV-1 latency. We then constructed the in vitro HSF1-knockout (HSF1-KO) HIV-1 latency models and found that HSF1 depletion inhibited the reactivation ability of LRAs including salubrinal, carfizomib, bortezomib, PR-957 and resveratrol, respectively. Furthermore, bortezomib/carfizomib treatment induced the increase of heat shock elements (HSEs) activity after HSF1-KO, suggesting that HSEs participated in reversing the latent HIV-1. Subsequent investigation showed that latent HIV-1-reversal by H_2_O_2_-induced DNA damage was inhibited by PARP1 inhibitors, while PARP1 was unable to down-regulate HSF1-depleted HSE activity, indicating that PARP1 could serve as a replaceable protein for HSF1 in HIV-1 latent cells. In summary, we succeeded in finding the mechanisms by which HSF1 reactivates the latent HIV-1, which also provides a theoretical basis for the further development of LRAs that specifically target HSF1.

## 1. Introduction

The spread of HIV-1 has been controlled effectively and patients’ life span has been prolonged since the Highly Active Anti-Retroviral Therapy (HAART) was applied in patients with Acquired Immune Deficiency Syndrome (AIDS) [1]. However, once the therapy is stopped, the viremia level will bounce back to the previous status in a very short time, which forces patients to keep taking medicine for their whole life [2]. HIV-1 latent reservoirs are the main reason why it is so difficult to cure HIV-1 [3]. Pre-latent HIV-1 in cells does not express its related RNAs or proteins, thus it cannot be eliminated by medicine or the immune system and continues to exist in patients’ bodies [4]. There has been increasing evidence that eliminating HIV-1 latent reservoirs could delay the spread of HIV-1 and even cure it effectively. One of the most effective strategies to fight against HIV-1 latent reservoirs is called “shock and kill” [5]. The strategy utilizes latent activators to activate latent HIV-1 and then eliminates the latent HIV-1 infected cells by medicine or the immune system, while the healthy CD4^+^ T cells are protected from viral infection. Eventually, HIV-1 latent reservoirs will be eliminated to achieve the functional cure of AIDS. A series of latent activators, named latency-reversing agent (LRAs), have been developed so far in terms of the formation mechanism of HIV-1 latent reservoirs. However, the development of these latent activators has been limited because of various problems including the inability to shrink latent reservoirs, poor diversity and side effects with virulence [6,7]. Therefore, the investigation of new therapeutic targets of HIV-1 latent reactivation and novel latent activators is particularly important for functional cure of HIV-1.

Heat shock factors 1 (HSF1) affects the expression of many genes at transcriptional level. It can interact with a specific DNA element which is the heat shock element (HSE) to modulate the transcription of heat shock proteins (HSPs) and some other related genes [8]. In 2011, Rawat et al. found that HSF1 could regulate HIV-1 gene transcription positively [9]. Our previous study also showed that HSF1 was probably an important transcriptional regulator in the process of latent HIV-1 reactivation, and that chemicals which stimulate HSF1 were likely to become new activators of latent HIV-1 [10]. The phosphorylated HSF1 would enter into the nucleus after stimulation by specific chemicals. The p300 protein which was recruited by HSF1 would further facilitate HSF1 acetylation and enrichment of the HIV-1 LTR, followed by the initiation of HIV-1 DNA transcription. Meanwhile, p-TEFb could be recruited by HSF1, leading to HIV-1 DNA transcriptional initiation and elongation. In addition, our further studies showed that HSF1 could promote the expression of downstream HSP90, thereby protecting p-TEFb subunit CDK9 from ubiquitination and degradation, which formed positive feedback of HSF-1-related signaling.

In this study, we investigated the possibility of HSF1 as an active target of latent HIV-1 reactivation. Our results showed that HSF1 was a crucial regulator during the reactivation of latent HIV-1. However, other regulators may be able to replace HSF1 and act on HSE to activate HIV-1 latent reservoirs upon HSF1 knockout. It has been known that DNA damage caused by cellular stress can lead to reactivation of latent HIV-1 such as with mitomycin C and UV [11]. In the meantime, X-ray treatment could possibly cause latent HIV-1 to reactivate while PARP1 participates in the DNA damage and repair pathways [12]. Further study suggested that PARP1 might play a role in the latent HIV-1 reactivation as protein substitutes for HSF1.

## 2. Materials and Methods

### 2.1. Cell Lines and Culture

J-Lat 10.6 cells (a Jurkat cell including HIV-1 full-length genome whose Env was frameshifted and inserted with GFP in place of Nef) and ACH2 cells (A3.01 cell integrated HIV-1 proviral DNA) were obtained from the National Institutes of Health AIDS Research and Reference Reagent Program. J-Lat 8.4 cells were another cell line with HIV-1 full length genome similar to J-Lat 10.6 cells. J-Lat NIB cells were generated by infecting Jurkat cells with pseudotyped NL4-3-ΔEnv/d2EGFP-Nef-IRES-Bcl2 (NIB) viruses. The above HIV-1 related cells were cultured in RPMI1640 (Gibco, Grand Island, NY, USA) with 10% fetal bovine serum (Capricorn, Ebsdorfergrund, Germany) and 1% penicillin/streptomycin solution (Gibco, USA). The TZM-bl cell line was obtained from the NIH AIDS reagent program. Both TZM-bl cells and HEK-293T cells were maintained in DMEM (Gibco) with 10% fetal bovine serum (Gemini Bio, West Sacramento, CA, USA) and 1% penicillin/streptomycin solution (Gibco). All cells were cultured in an incubator containing 37 °C and 5% CO_2_.

### 2.2. Reagent

Carfizomib (S2853), JQ1 (S7110), salubrinal (S2923), PR-957 and bortezomib (S1013) were purchased from Selleck Chemicals (Houston, TX, USA). SAHA, prostratin were purchased from Sigma-Aldrich (St. Louis, MO, USA). Resveratrol was purchased from MedChem Express (Monmouth Junction, NJ, USA). TNF-α was purchased from R&D Systems. Antibodies specific to HSF1 and β-actin were from Cell Signaling Technology (Beverly, MA, USA). Antibodies specific to PARP1 were from Santa Cruz Biotechnology (Santa Cruz, CA, USA). Antibodies specific to p24 (183-12H-5C) were obtained from the National Institutes of Health AIDS Research and Reference Reagent Program. Plasmid vector LentiCRISPRv2, pCDH, pVSVg and psPAX2 were obtained from Addgene. PolyJet was purchased from SignaGen Laboratories (SL100688, USA). Puromycin was purchased from VWR-AMRESCO (USA).

### 2.3. HSF1 Knockout Assay

Editable gRNAs were designed by Zhang Lab Guide Design Resources website. The CRISPRv2 plasmid construction was according to the manufacture’s protocol using the following oligos (gRNA1) 5′-AGATGAGCGCGTCGGTGTCC-3′, which targets exon 1 of HSF1. gRNA2 oligos: 5′-CAGATGAGCGCGTCGGTGTC-3′, which targets exon 1 of HSF1. gRNA3 oligos: 5′-ACTGGCCCTGGTCGAACACG-3′, which targets exon 2 of HSF1. gRNA4 oligos: 5′-GTTGAGCTGCCGCACGAAGC-3′ which targets exon 2 of HSF1. gRNA5 oligos: 5′-CATGTCGGGCACGGTCACCG-3′ which targets exon 11 of HSF1.The plasmid vector LentiCRISPRv2, pVSVg and psPAX2 were co-transfected into HEK-293T cells by using PolyJet. Two days post transfection, the lentivirus was collected and the J-Lat 10.6 cells were infected by lentivirus with Polybrene. Two days after infection, cells were exposed to 1.5 μg/mL puromycin. The drug-resistant cells were collected and sorted as single colonies by MoFlo XDP (Beckman Coulter, Brea, CA, USA). The expression of HSF1 was detected by Western blotting. The HSF1 KO monoclonal cell line was confirmed using Polymerase Chain Reaction (PCR) assay and the following primers (gRNA1): forward primer, 5′-CGCCTATTCCCTCCTTGCTC-3′, reverse primer, 5′-AGCCCAATACAAGAGACGCC-3′.

### 2.4. HSF1 Knockdown Assay

For the knockdown of HSF1, negative control siRNA (siNC) was purchased from RiboBio (Guangzhou, China). siHSF1-1, siHSF1-2 and siHSF1-3 were used as a mixture and have been validated by the company to ensure that at least one siRNA was able to knock down HSF1 up to 75%. HSF1 knockdown with siRNAs was used in a heterogeneous latency model J-Lat NIB to further understand the role of HSF1 on HIV-1 latency reactivation. Each gene was knocked down by three different siRNAs. For the suspension cell J-Lat NIB cell line, nucleotransfection of the siRNA used nucleofector solution (Lonza, Basel, Switzerland) according to the manufacturer’s instructions. The knockdown efficiency was confirmed by Western blot.

### 2.5. Flow Cytometry

WT cells (J-Lat 10.6 cells), NC cells (J-Lat 10.6 cells infected with lentivirus targeting dummy guide) and KO cells (J-Lat 10.6 cells infected with lentivirus and knockout HSF1) were treated with the indicated compounds in 48-well plates, J-Lat NIB cells were treated with LRAs after transfection with siRNAs or HSF1 inhibitor KRIBB11 and J-Lat 8.4 cells were also treated with KRIBB11, collected and washed after 48 h. GFP expression was analyzed by BD FACS Canto II (San Jose, CA, USA). All the data were analyzed using FlowJo V10 software. The rate of GFP positive cells within the whole population indicated the levels of HIV-1 reactivation.

### 2.6. Western Blotting Analysis

After treatment, collection and washing with PBS, the cells were exposed to the pre-frozen RIPA lysis buffer with phosphatase and protease inhibitors (KeyGen BioTECH, JS, CHN) on ice for 15 min and vortexed well. The supernatant was collected in a new pre-frozen tube after the lysates were centrifuged at 12,000× *g* for 15 min at 4 °C. About 60–120 μg proteins were separated on 10% SDS-polyacrylamide gel. Then, the separated protein was transferred onto a PVDF membrane (Roche, Indianapolis, IN, USA). The PVDF membranes were immersed with primary antibodies after blocking for 1 h with 5% nonfatty milk solution. After 8 h incubation at 4 °C, the membranes were washed and incubated with homologous secondary antibodies for 1 h. Subsequently, the membranes were exposed to FlourChem E Systerm (Protein Simple, San Jose, CA, USA) with ECL (Millipore, Burlington, MA, USA).

### 2.7. Construction of 293T-HSE-Luc Cells

HSE sequences were obtained from Thomas’s description [13], and 6 HSEs derived luciferase genes were cloned into pCDH plasmid. The following is the 6HSE-luciferase sequence: CTCGAGAACGTTCTAGAACGTCGAGAACGTTCTAGAACGTCGAGAACGTTCTAGAACGTCGAGAACGTTCTAGAACGTCGAGAACGTTCTAGAACGTCGAGAACGTTCTAGAAC+TATA+luciferase. Then, we co-transfected the pcDH-6HSE-luciferase, psPAX2 and pVSVg into HEK-293T cells, collected the supernatant after 48 h and infected the HEK-293T cells. These infected cells were screened with 2 μg/mL puromycin and we obtained the monoclonal cell line named 293-HSE-Luc cells. After HSF1 Knockout Assay, we obtained knock-down cell lines named 293-HSE-Luc-HSF1^KD^3 cells (gRNA 3) and 293-HSE-Luc-HSF1^KD^5 (gRNA5). Monoclonal cell lines were obtained by limiting dilution analysis, termed 293-HSE-Luc^△^^HSF^^1^3 (from 293-HSE-Luc-HSF1^KD^3) and 293-HSE-Luc^△^^HSF^^1^5 (from 293-HSE-Luc-HSF1^KD^5).

### 2.8. Luciferase Assays

Typically, cells were evenly seeded into a 96-well plate and treated with a series of concentrations of the LRAs for 48 h. Cells were removed from the supernatant and washed with PBS, followed by lysing with 50 μL of 1× lysis reagent (Promega, Madison, WI, USA). Then, 25 μL of the lysate was transferred to the white plate after 15 min shaking, detecting the expression of luciferase with the same volume of luciferase substrate (Promega, USA) by Infinite M1000 pro (Tecan, Männedorf, Switzerland).

### 2.9. RT-qPCR

Total mRNA from cells was isolated using the Cellular total RNA isolation Kit (Foregene, China) followed by reverse transcription using PrimeScript RT mix (Takara, Japan). Real-Time PCR (Promega, USA) was executed on Light Cycler 480 Instrument (Roche, Switzerland) using the following primers: HIV-1 Gag gene forward primer: 5′-GTCCAGAATGCGAACCCAGA-3′, reverse primer: 5′-GTTACGTGCTGGCTCATTGC-3′. HIV-1 Tat gene forward primer: 5′-ATGGAGCCAGTAGATCCTAGACT-3′, reverse primer: 5′-CGCTTCTTCCTGCCATAGGA-3′. HIV-1 Vif gene forward primer: 5′-CACACAAGTAGACCCTGACCT-3′, reverse primer: 5′-CCTACCTTGTTATGTCCTGCT-3′. HIV-1 Vpr gene forward primer: 5′-CCACAAAGGGAGCCATACAATG-3′, reverse primer: 5′-TTATGGCTTCCACTCCTGCC-3′. HIV total RNA forward primer: 5′-CTGGCTAACTAGGGAACCCACTGCT-3′, reverse primer: 5′-GCTTCAGCAAGCCGAGTCCTGCGTC-3′ [14]. GAPDH gene as the reference for normalization and the forward primer: 5′-CTCTGCTCCTCCTGTTCGAC-3′, reverse primer: 5′-AGTTAAAAGCAGCCCTGGTGA-3′. The analysis of mRNA enrichment used the 2^−ΔΔCp^ method.

### 2.10. Statistical Analysis

All the experimental results were represented as the mean ± SD value of three independent tests. The analysis software GraphPad Prism 8.0 (San Diego, CA, USA) was used to perform statistical analysis of comparisons between groups using one-way ANOVA test followed by Tukey’s *t*-test. *p* values less than 0.05 were considered statistically significant. *p* values were defined as * p <0.05, ** p <0.01, *** p <0.001.

## 3. Results

### 3.1. LRAs Reactivation Activity was Reversed after HSF1 Knockout

To study the role of HSF1 on the latent HIV-1 reservoir reactivation, we generated a J-Lat 10.6 HSF1 knockout cell line by lentivirus method. The J-Lat 10.6 cell line was an HIV-1 reservoir cell line which contained the frameshift inactivation of Env and the GFP gene replacing the viral Nef. Thus, the percentage of GFP-positive cells could implicate the degree of HIV-1 reservoir reactivation. The negative control (NC) cell line comprised J-Lat 10.6 cells which were infected with blank vector lentivirus. As shown in Figure S1, HSF1 protein was completely silent in the J-Lat 10.6 HSF1 knockout cell line, in which 2 bp and 1 bp base was inserted in sister chromatids, respectively (Figure S1A,B).

**Figure 1:**
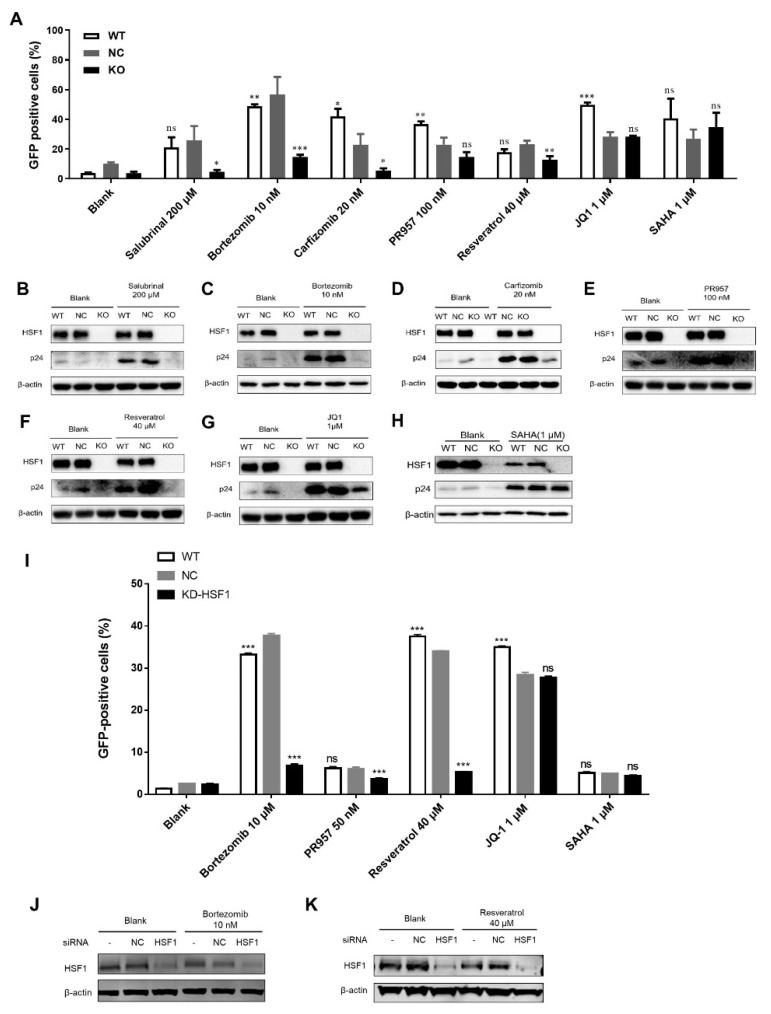
LRAs’ reactivation activity was reversed after HSF1 knockout**.** (**A**–**H**) WT, NC and HSF1-KO J-Lat 10.6 cell lines were treated with LRAs within 48 h and GFP-positive cells percentages were evaluated by flow cytometry (**A**) and Western blot against p24 proteins (**B**–**H**). (**I**–**K**) The GFP-positive percentages of J-Lat NIB cells upon knockdown of HSF1 and treatment of different LRAs were evaluated by flow cytometry (**I**). The knockdown efficiencies of HSF1 were evaluated by Western blot against HSF1 proteins (**J**,**K**). The *p*-value was defined as * *p* < 0.05, ** *p* < 0.01 and *** *p* < 0.001 vs. control; ns was no statistical significance.

Classical LRAs that reactivated HIV-1 reservoirs by targeting different cellular pathways, such as JQ1 and SAHA, as well as chemicals reported to reactivate HIV-1 reservoirs through activating HSF1, such as salubrinal, bortezomib, carfizomib [10], PR-957 [15] and resveratrol [16], were used to treat wildtype (WT) cells, NC cells, and HSF1 KO cells for 48 h. As shown in Figure 1A, the percentage of GFP-positive cells in the untreated WT cell was less than 4%, while for the untreated NC cells the percentage was about 9%, and for the untreated KO cells it was less than 2%, which indicated that the expression of HIV-1 proviral DNA in unstimulated J-Lat 10.6 cells tended to be lower after HSF1 knockout (Figure 1A). After treatment with salubrinal (200 μM), bortezomib (10 nM), carfizomib (20 nM), and resveratrol (40 μM), the expression of GFP-positive cells in KO groups was blocked compared with that in WT and NC groups. However, the expression of GFP-positive cells in the KO cells treated with PR-957 did not decrease significantly but still showed the downward trend which was caused by its additional upregulation of p-TEFb expression. In contrast, the JQ1 (1 μM) and SAHA (1 μM) could still activate the latency HIV-1 in KO cells. We further detected the expression level of HIV-1 particles p24 protein in the cells (Figure 1B–H). The results showed that the p24 expression in WT and NC groups was significantly higher than that in the KO group after treatment with salubrinal, bortezomib, carfizomib, PR-957, and resveratrol, which was similar to the percentages of GFP-positive cells. However, there was no significant difference in the expression of p24 protein among the three groups which were treated with JQ1 and SAHA. Taken together, these results indicated that HSF1 was involved in the reactivation process of HIV-1 reservoirs.

**Figure 2:**
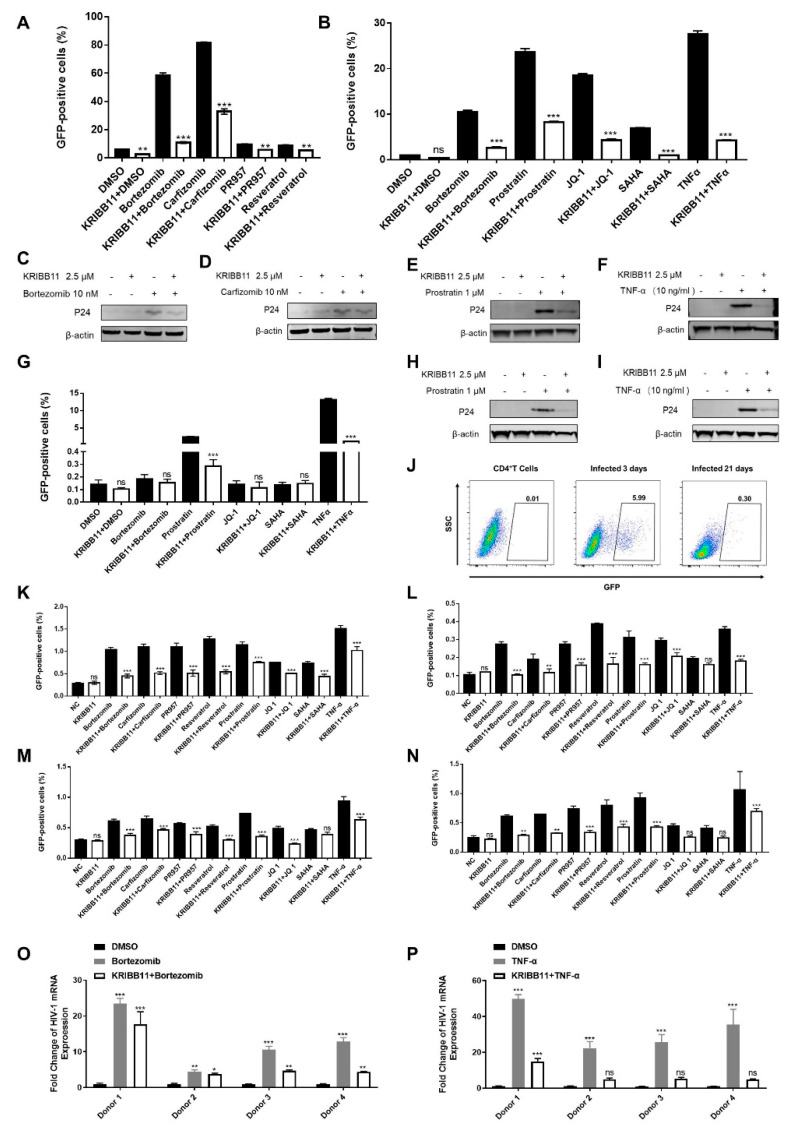
LRAs reactivation activity was reversed by inhibitor of HSF1**.** (**A**–**F**) The GFP-positive percentages of J-Lat NIB cells were treated with HSF1 inhibitor KRIBB11, followed with different LRAs. The GFP-positive percentages of J-Lat NIB cells were evaluated by flow cytometry (**A**,**B**). The reactivation efficiencies within different groups were also evaluated by Western blot against p24 proteins (**C**–**F**). (**G**–**I**) J-Lat 8.4 cells were treated with LRAs with/without KRIBB11. The GFP-positive percentages of J-Lat 8.4 cells were evaluated by flow cytometry (**G**) and p24 proteins were evaluated by Western blot (**H**,**I**). Latently HIV-1 primary infection cells construction process (**J**). (**K**–**P**) The latently HIV-1 primary infection cells were treated with HSF1 inhibitor KRIBB11, followed by treating with different LRAs. The GFP-positive percentages of latently HIV-1 primary infection cells were evaluated by flow cytometry. Data showed results from four different healthy donors. (**K**) Donor 1, (**L**) Donor 2, (**M**) Donor 1, (**N**) Donor 4. (**O**,**P**) The reactivation efficiency within bortezomib and TNF-α were validated by the amount of intracellular HIV-1 total RNAs. The *p*-value was defined as * *p* < 0.05, ** *p* < 0.01 and *** *p* < 0.001 vs. control; ns was no statistical significance.

The J-Lat 10.6 cell line is a monoclonal latency cell line [17]. To further elucidate the contribution of HSF1 on the reactivation of HIV-1 latency, we knocked down HSF1 in a heterogeneous latency model J-Lat NIB [18]. We found that the depletion of HSF1 significantly decreased the reactivation efficiencies of several LRAs including bortezomib, PR-957, resveratrol, JQ1 and SAHA (Figure 1I–K). We also treated J-Lat NIB cells and J-Lat 8.4 cells with an HSF1 inhibitor named KRIBB11 [19]. The results showed that the HIV-1 latency-reversing activities of LRAs were significantly decreased upon inhibiting HSF1 (Figure 2A,B,G). The inhibition of HIV-1 latency reactivation was also confirmed by Western blot against HIV-1 p24 proteins (Figure 2C–F,H,I).

To investigate whether HSF1 targeted HIV-1 promoter activity directly, we conducted further experiments in an HIV-1 expression cell line, TZM-bl cells [20]. The expression of luciferase which was under the control of HIV-1 LTR could indicate the activity of an HIV-1 promoter. Firstly, we overexpressed HSF1 in TZM-bl cells, followed by co-treating with different LRAs. The results showed that the overexpression of HSF1 was able to enhance the reactivation activities of LRAs (Figure S1C). Secondly, we knocked out the endogenous HSF1 by CRISPR/CAS9 technology in TZM-bl cells, followed by the treatment of LRAs. The results showed that the depletion of HSF1 also significantly restricted LRAs-mediated HIV-1 expression (Figure S1D). The re-expression of HSF1 in HSF1-KO TZM-bl cells was able to restore the activity of the HIV-1 promoter, while the co-treatment of KRIBB11 inhibited HSF1-mediated restoration (Figure S1E). We also co-treated wildtype TZM-bl cells with KRIBB11 and different LRAs as control. Our results showed that the inhibition of HSF1 by KRIBB11 was able to significantly inhibit LRAs-mediated activation of HIV-1 promoter (Figure S1F).

We further established an HIV-1 latent model in primary CD4+ T cells. The GFP expression level was detected 3 days after infection. The Donor 1 infection efficiency reached 5.99% and it was confirmed that most of the GFP-positive cells had entered into latency at the 3rd week (Figure 2J). We treated those cells with LRAs and KRIBB11 for 2 days. It was found that the HIV-1 latency reactivation of LRAs was observably reduced by inhibiting HSF1 (Figure 2K–N) and the inhibition of HIV-1 reactivation efficiency was confirmed by qPCR. (Figure 2O,P).

Overall, our above results indicated that HSF1 contributed to the reactivation of HIV-1 latency by targeting the HIV-1 promoter directly.

### 3.2. LRAs Promoted the Binding of HSF1 to HSE

**Figure 3:**
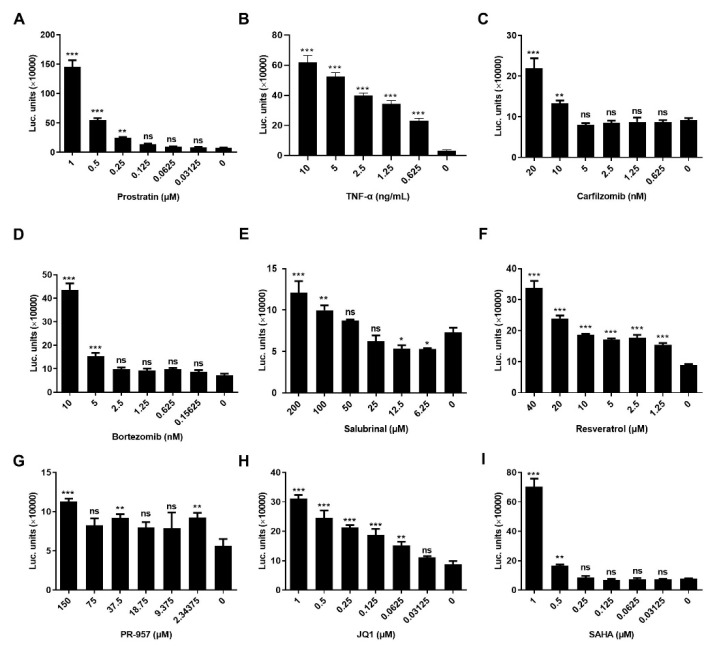
LRAs promoted the binding of HSF1 to HSE**.** 293-HSE-Luc cells were treated with different concentrations of prostratin (**A**), TNF-α (**B**), carfizomib (**C**), bortezomib (**D**), Salubrinal (**E**), resveratrol (**F**), PR-957 (**G**), JQ1 (**H**) and SAHA (**I**) for 48 h and the luciferase activities were evaluated. All data were reported from three independent experiments as the mean ± SEM. The *p*-value was defined as * *p* < 0.05, ** *p* < 0.01 and *** *p* < 0.001 vs. control; ns was no statistical significance.

In order to explore how HSF1 stimulated the expression of HIV-1 LTR in cells after LRAs treatment, we overexpressed the luciferase driven by the heat shock element (HSE) in HEK-293T cells to obtain monoclonal cell line 293-HSE-Luc. The HSE DNA motif is the binding site of HSF1, which is composed of at least three inverted repeated nGAAn sequences, and the continuous HSE sequences have a stronger binding affinity to HSF1 proteins. When HSF1 proteins form a trimer and merge into the nucleus, they undergo phosphorylation, acetylation and SUMOylation modifications. These activated HSF1 proteins interact with HSE on the HSE-luciferase sequence which is integrated into the genome to promote the transcription of downstream luciferase genes. The results showed that all the LRAs could significantly increase the luciferase expression (Figure 3). LRAs stimulated the expression of luciferase in a concentration-dependent manner. These data indicated that these LRAs could promote the binding of HSF1 to HSE.

### 3.3. LRAs Changed HSE Activity after HSF1 Deletion

**Figure 4:**
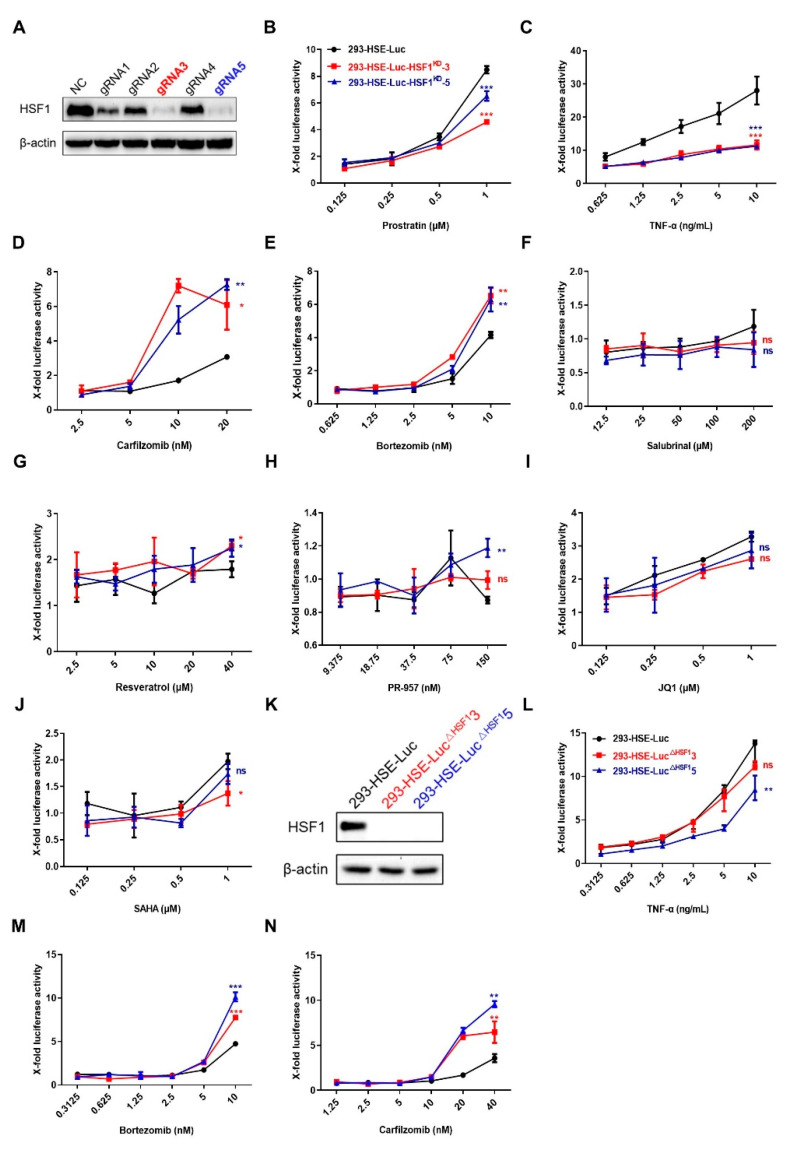
LRAs changed HSE activity after HSF1 deletion**.** (**A**) The efficiencies of HSF1 depletion in 293-HSE-Luc cell line were evaluated by Western blot. (**B**–**J**) WT and knockdown cell lines were treated with different LRAs for 48 h. The expression of luciferase within different groups in each time point was evaluated. The treatment of prostratin and TNF-α showed a lower luciferase expression upon knocking down HSF1 (**B**,**C**). Carfizomib and bortezomib treatment showed a higher luciferase expression upon knocking down HSF1 (**D**,**E**). Salubrinal, resveratrol and PR-957 treatment did not induce significant changes of luciferase expression upon depleting HSF1 (**F**–**H**). The treatment of JQ1 and SAHA induced slight decrease of luciferase expression upon knocking down HSF1 (**I**,**J**). (**K**) The knockout efficiencies of two monoclonal cell lines were evaluated by Western blot. (**L**–**N**) The fold change of luciferase expression in WT and HSF1-KO monoclonal cell lines were evaluated upon co-treating with different LRAs. All data were reported from three independent experiments as the mean ± SEM and the *p*-value was defined as * *p* < 0.05, ** *p* < 0.01 and *** *p* < 0.001 vs. control; ns was not statistically significant.

To further verify whether the LRAs could still influence the response of HSE in the absence of HSF1, we knocked down HSF1 in 293-HSE-Luc cells and successfully gained two knockdown cells (Figure 4A). As shown in Figure 4B–J, the fold change of luciferase expression in the knockdown group was lower than that in the NC group after treatment with prostratin, SAHA, JQ1 and TNF-α, while the fold change of luciferase expression in the knockdown group treated with bortezomib and carfizomib, which were reported to reactivate latent HIV-1 by activating HSF1, was higher than that in the NC group (Figure 4B–E). However, there was no obvious change after salubrinal, resveratrol and PR-957 treatment (Figure 4F–H). Furthermore, we also obtained the similar results in HSF1 knockout monoclonal cell lines (Figure 4K–N).

Therefore, we speculated that there might be a regulator substituting for HSF1, and its binding to HSE would contribute to the expression of luciferase in cells with HSF1 depletion. The LRAs have different selectivity for this substitute protein than for HSE. Those LRAs which were clearly reported to reactivate HIV-1 reservoir by HSF1 were more selective for substitute proteins binding to HSE, leading to a higher HSE-driven luciferase increase.

### 3.4. PARP1 Acted as HSF1 Potential Substitute Protein to Promote the Reactivation of HIV-1 Reservoir

The poly ADP-ribose polymerase 1 (PARP1) is involved in cellular DNA repair, chromosome structure and transcription repair [21]. HSF1 can recruit PARP1 through the scaffold protein PARP13. These proteins can form a synergistic complex which promotes the binding of HSF1 to its binding site and initiates gene transcription [22,23,24]. During the heat shock response, HSF1 proteins within the complex firstly bind to HSE, which triggers PARP1 activation and HSF1-PARP13 separation. HSF1-PARP13 promotes the location and binding of PARP1 to those DNA damage sites. At the same time, inhibiting PARP1 can increase the level of Siah1 to promote the ubiquitination of Ell2 protein, which can further inhibit the function of p-TEFb and inhibit HIV-1 transcription [25]. Proteasome inhibitors such as bortezomib and carfizomib can stabilize the Ell2 protein by inhibiting the action of the proteasome and promote the reactivation of latent HIV-1 [26]. Therefore, PARP1 may play a certain role in the function of HSF1 in the process of HIV-1 reservoir reactivation.

**Figure 5:**
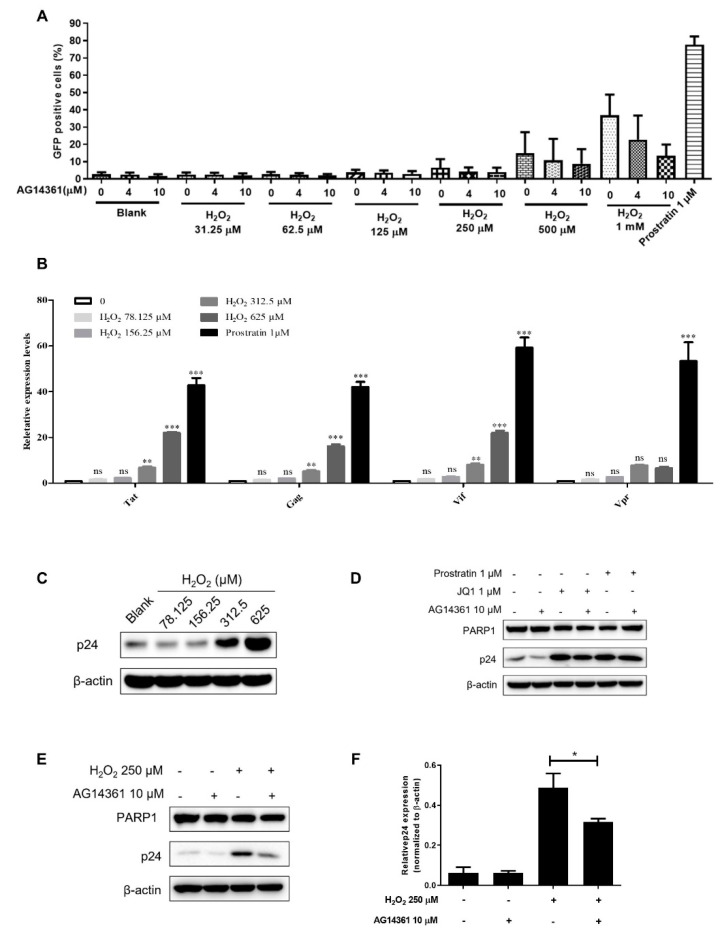
PARP1 acted as HSF1 potential substitute protein to promote the reactivation of HIV-1 reservoir. (**A**) J-Lat 10.6 cells were treated with different concentrations of H_2_O_2_, ranging from 31.25 μM to 1 mM. Both 4 μM and 10μM of AG14361 were also utilized to treat these cells. About 48 h post treatment, GFP-positive cells within different groups were evaluated by flow cytometry. (**B**) J-Lat 10.6 cells were treated with different concentrations of H_2_O_2_, ranging from 78.125 μM to 625 μM. The expression of HIV-1 HIV-1 *Tat*, *Gag*, *Vif* and *Vpr* mRNA was quantitated by RT-qPCR. (**C**) ACH2 cells were treated with different concentrations of H_2_O_2_. The expression of HIV-1 was evaluated by Western blot against HIV-1 p24 proteins. (**D**) ACH2 cells were co-treated with prostratin, JQ-1, and/or AG14361. The expression of PARP1 and HIV-1 p24 proteins was evaluated by Western blot. (**E**,**F**) ACH2 cells were co-treated with 250 μM H_2_O_2_ and/or 10 μM AG14361. The expression of PARP1 and p24 proteins was evaluated by Western blot (**E**). The expression of p24 proteins was quantitated with Image J software (**F**). All data were reported from three independent experiments as the mean± SEM and the *p*-value was defined as * *p* < 0.05, ** *p* < 0.01 and *** *p* < 0.001 vs. control; ns was no statistical significance.

In order to verify whether the activation of PARP1 could lead to the activation of latent HIV-1, we used H_2_O_2_ to induce DNA damage and then detected the percentage of GFP-positive cells in J-Lat 10.6 cells. The results show that H_2_O_2_ could reactivate HIV-1 reservoirs to 6.60% and 14.97% at the concentration of 250 μM and 500 μM, respectively (Figure 5A). However, the increased percentage of GFP-positive cells was reversed in the presence of PARP1 inhibitor AG14361. Similarly, compared with the blank control, the mRNA expression of HIV-1 related genes in J-Lat 10.6 cells which were treated with H_2_O_2_ was increased significantly (Figure 5B). This indicated that DNA damage induced by H_2_O_2_ had a tendency to promote the reactivation of HIV-1 reservoirs, while the inhibition of PARP1 could reverse this trend. Furthermore, we detected the changes of HIV-1 capsid protein p24 in another HIV-1 latency cell line, ACH2 cells. Compared with the blank group, H_2_O_2_ could increase the expression of HIV-1 p24 protein at 312.5 μM (Figure 5C) and the upregulated p24 expression could be reversed by AG14361 (Figure 5D–F). However, AG14361 was unable to inhibit the expression of HIV-1 p24 which was induced by prostratin and JQ1 (Figure 5D). Therefore, PARP1 was positively correlated with the reactivation potential of latent HIV-1. 

**Figure 6:**
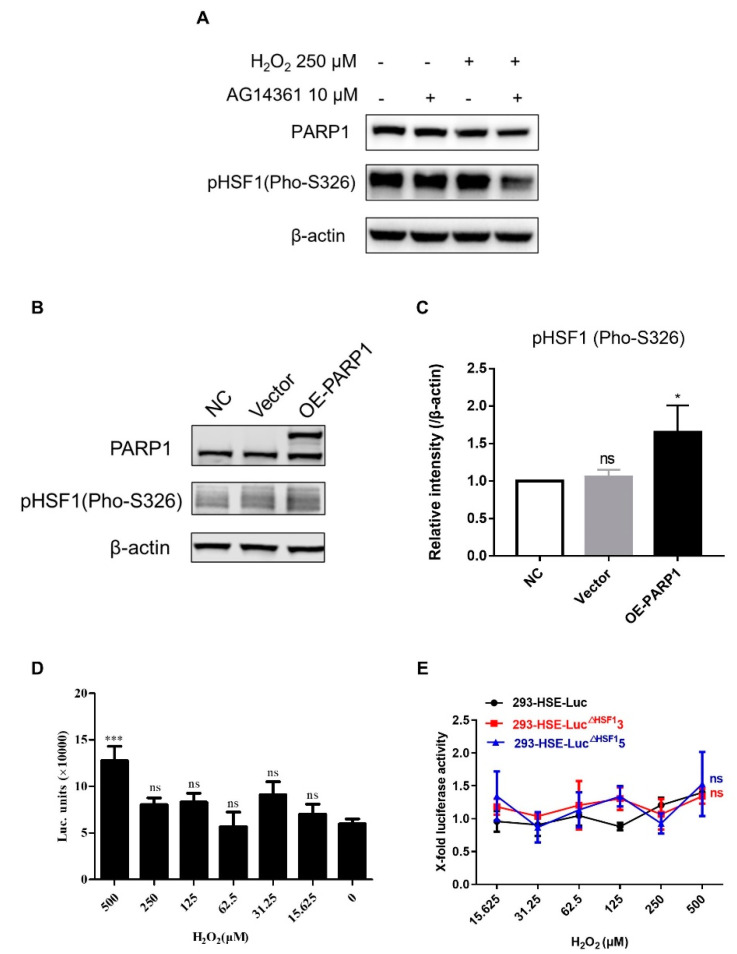
PARP1 was closely related to HSF1 in latent reactivation**.** (**A**) ACH2 cells were treated with H_2_O_2_ (250 μM) and AG14361 (10 μM) for 48 h and the expression of PARP1 and pHSF1 (Pho-326) was evaluated by Western blot. (**B**,**C**) PARP1 was overexpressed in HEK293T cells. The expression of PARP1 and pHSF1 (Pho-S326) proteins was evaluated by Western blot (**B**). The expression of pHSF1 was quantitated with Image J software (**C**). (**D**,**E**) WT,293-HSE-Luc^Δ^^HSF1^3 and 293-HSE-Luc^Δ^^HSF1^5 cells were treated with various concentrations of H_2_O_2_, ranging from 15.625 μM to 500 μM. The expression of luciferase was monitored in WT group (**D**) and HSF1-KO groups (**E**). All data were reported from three independent experiments as the mean± SEM and the *p*-value was defined as * *p* < 0.05, and *** *p* < 0.001 vs. control; ns was no statistical significance.

To further verify the correlation between PARP1 and HSF1, we evaluated the expression level of phosphorylated HSF1 (pHSF1) proteins after the combined treatment of H_2_O_2_ and AG14361. We found that the expression of pHSF1 in H_2_O_2_ and AG14361 co-treated cells was significantly downregulated compared with that in the cells treated with H_2_O_2_ only (Figure 6A) and overexpression of PARP1 increased the phosphorylation of HSF1 on HEK-293T cells (Figure 6B,C). Luciferase expression was induced by H_2_O_2_ in 293T-HSE-Luc cells at the concentration of 500 μM, while expression was completely reversed by HSF1 depletion (Figure 6D,E). These results indicated that PARP1 was closely related to HSF1 in latent reactivation.

## 4. Discussion

Currently, flat-tailed macaques can form a stable HIV-1 latent reservoir after infection with HIV-1. This animal model can be used to evaluate the activity of LRAs in vivo and contribute to screening for effective LRAs used in clinics [27]. Although there are some LRAs being explored in clinical trials, some defects including the inability to reduce the HIV-1 reservoir, poor specificity and high toxicity have hindered them from being further utilized clinically [28,29]. Therefore, to develop more potential LRAs, searching for new targets for HIV-1 reservoir reactivation has become a new research priority.

The heat shock response is a highly conservative defense adaptation response of the body, which is characterized by changes in gene expression in response to heat stress [30]. HSF1 is closely related to the HIV-1 life cycle. In the early stage of HIV-1 infection, HSF1 can promote the replication of HIV-1 and the expression of viral genes. It also regulates the expression of other downstream heat shock proteins [9,31,32,33]. The restriction of HSF1 activation can reduce the infectivity and total quantity of HIV-1, which may be one of the early conditions for the HIV-1 latency formation [34]. Our previous research found that HSF1 was positively correlated with the reactivation of the HIV-1 reservoir [10]. HSF1 enters into the nucleus after phosphorylation, and at the same time it recruits p300 to promote its own acetylation and further binds to the HIV-1 LTR. Meanwhile, HSF1 also recruits p-TEFb to promote HIV-1 transcriptional elongation. Further studies showed that HSF1 could promote the expression of downstream protein HSP90, which protects p-TEFb subunit CDK9 from ubiquitination and degradation, and moderates the effect of HSF1 with positive feedback loops. Since HSF1 plays an important role in latent HIV-1 reactivation, chemicals targeting HSF1 are highly likely to become new LRAs. The LRAs which were discovered to reactivate HIV-1 reservoirs through activating HSF1 might have a relatively high cytotoxicity due to the unselective inhibition on the proteasome [15,16,35]. Therefore, safer and more efficient LRAs activating HSF1 are urgently needed.

In recent years, studies on HSF1-mediated HIV-1 reservoir reactivation have been reported by some groups. Peng et al. found that thiostrepton could reactivate HIV-1 reservoir through enriching p-TEFb and NF-κB which were recruited by HSF1 pathway [35]. Timmons et al. used HSF1 inhibitor KRIBB11 to inhibit HSF1 and found that inhibiting HSF1 could attenuate the reversal of HIV-1 latency by LRAs including histone deacetylase inhibitors (HDACi), protein kinase C (PKC) agonists, and proteasome inhibitor [19]. However, we found that HSF1 knockout in the HIV-1 latency cell line did not effectively attenuate the reversal of HIV-1 latency mediated by HDACi (SAHA) and the proteasome inhibitor still works.

In this study, we edited HSF1 in the HIV-1 reservoir cell line J-Lat 10.6 to confirm its role in the reactivation of HIV-1 reservoir. In the detection of the reactivation effect of different LRAs on knockout cell lines, it was found that the activation effect of TNF-α, prostratin, JQ1 and SAHA did not change compared with that of HSF1 non-knockout cells. These LRAs have been confirmed to activate the HIV-1 reservoir through different pathways. TNF-α activates global T cells, while prostratin promotes the binding of transcription factors NF-κB and Ap-1 to the HIV-1 LTR through phosphorylation of PKC. SAHA inhibits HDAC activities, and JQ1 promotes the binding of p-TEFb to Tat protein by hijacking BRD4 proteins. However, carfizomib, bortezomib, salubrinal can effectively reactivate the HIV-1 reservoir in non-knockout HSF1 cells, while their activation effect decreases in knockout HSF1 cells. The reason may be due to their direct promotion effects on the phosphorylation of HSF1 and its binding with HSE on the HIV-1 LTR. Combined with the results of previous research, we confirm that HSF1 has the potential to be a new HIV-1 latent activation drug target, although there may be other factors involved in the reactivation of the HIV-1 reservoir.

Under the stimulation of stress conditions, HSF1 forms a trimer after phosphorylation, which facilitates HSF1 entering into the nucleus and binding to HSE to regulate gene transcription [36]. Our research also confirmed that LRAs could effectively promote the binding of HSF1 to HSE using HSE-luciferase cells. Surprisingly, HSE activity increased when the HSF1-KD or HSF1-KO cells were treated with the LRAs that effectively reactivate HIV-1 reservoirs through activating HSF1 (salubrinal, bortezomib, carfizomib and resveratrol), but decreased in the cells treated with those activating the HIV-1 reservoir not mainly through HSF1 (prostratin, JQ1, SAHA and TNF-α). Therefore, there might be an unknown protein which could promote the increase of HSE activity when HSF1 protein is knocked down or knocked out. We speculate that a HSF1 family protein such as HSF2, HSF3, HSF4 or HSF1 chaperone proteins might play the role of HSF1 when HSF1 is absent.

HSF is an important transcription factor in cells, which can be used to resist stress response to cell damage. In the mammalian HSF family, HSF1 is the main transcription factor. HSF2 and HSF4 also exist in mammalian cells, and the DNA binding domain of the HSF family is highly conserved [37,38,39]. Transcription factors of the HSF family have a preference for binding to different types of HSE, among which HSF1 binds to continuous HSE, and HSF2 mainly binds to discontinuous HSE, while HSF4 can be combined with two types of HSE [40,41]. We found that knocking out HSF1 did not change the extensive reactivation of HIV-1 in J-Lat 10.6 cells. Therefore, we speculated whether other members of the HSF family are also involved the activation of the HIV-1 reservoir after HSF1 knockout. We thus tested the expression levels of other HSF proteins in HSF1-KO and WT cells. We found that HSF2, but not HSF4, was significantly increased in HSF1-KO cells (Figure S2A). There is currently no effective HSF2 inhibitor or activator, so CRISPR/Cas9 technology was used to knock out HSF2 within cells. However, the cells were unable to survive the subsequent culture after the HSF2 knocking out, hindering further research (Figure S2B).

PARP1 can promote HSF1 binding to its binding site, and the PARP1-HSF1 complex binding to HSE can mediate the localization of PARP1 to the DNA damage site. Studies have shown that PARP1 is related to the transcriptional regulation of HIV-1 [25]. DNA damage can promote HIV gene transcription. It was shown in our research that H_2_O_2_, which could cause DNA damage, could reactivate HIV-1 reservoirs, and the reactivation effect could be inhibited by PARP1 inhibitor AG14361, which means that PARP1 is correlated with latent HIV-1 reactivation by DNA damage. In addition, it has been reported that the treatment of DNA-damaging agents such as mitomycin C and UV can promote the expression of LTR-driven chloramphenicol acetyltransferase (CAT) [11]. Meanwhile, treatment using X-ray irradiation could also activate the expression of latent HIV-1, which seems to be involved in the p53 and the apoptotic pathways [12]. However, the reactivation effects of HIV-1 reservoirs caused by prostratin and JQ1 could not be inhibited by AG14361, which indicated that activating PARP1 is not involved in their reactivation effect on HIV-1 latency. It was further found that AG14361 could lead to the reduction of HSF1 phosphorylation, and that H_2_O_2_ could promote the activity of HSE, which was not affected in HSF1 knock-out cells. Taken together, these results indicate that PARP1 is closely related to HSF1 in latent HIV-1 reactivation. Therefore, PARP1 could substitute HSF1 and bind to HSE when HSF1 was knocked out.

In summary, HSF1 can be used as a drug target for new HIV-1 latency reactivators. PARP1 can act as a potential HSF1 substitution protein to stimulate the reactivation of HIV reservoirs.

## Data Availability

Not applicable.

## Supplementary Materials

The following are available online at https://www.mdpi.com/article/10.3390/cells11152331/s1, Figure S1. The depletion of HSF1 inhibited the HIV-1 promoter activity. Figure S2. The expression of HSF2 was increased upon HSF1 knockout.

## References

[1] Klaus B.D., Grodesky M.J. (1997). HIV and HAART in 1997. Highly active antiretroviral therapy. Nurse Pract..

[2] Vandamme A.M., Van Vaerenbergh K., De Clercq E. (1998). Anti-human immunodeficiency virus drug combination strategies. Antivir. Chem. Chemother..

[3] Finzi D., Hermankova M., Pierson T., Carruth L.M., Buck C., Chaisson R.E., Quinn T.C., Chadwick K., Margolick J., Brookmeyer R. (1997). Identification of a reservoir for HIV-1 in patients on highly active antiretroviral therapy. Science.

[4] Pierson T., McArthur J., Siliciano R.F. (2000). Reservoirs for HIV-1: Mechanisms for viral persistence in the presence of antiviral immune responses and antiretroviral therapy. Annu. Rev. Immunol..

[5] Deeks S.G. (2012). HIV: Shock and kill. Nature.

[6] Zhao M., De Crignis E., Rokx C., Verbon A., van Gelder T., Mahmoudi T., Katsikis P.D., Mueller Y.M. (2019). T cell toxicity of HIV latency reversing agents. Pharmacol. Res..

[7] Delagreverie H.M., Delaugerre C., Lewin S.R., Deeks S.G., Li J.Z. (2016). Ongoing Clinical Trials of Human Immunodeficiency Virus Latency-Reversing and Immunomodulatory Agents. Open Forum Infect. Dis..

[8] Feng H., Wang S., Guo L., Punekar A.S., Ladenstein R., Wang D.C., Liu W. (2016). MD simulation of high-resolution X-ray structures reveals post-translational modification dependent conformational changes in HSF-DNA interaction. Protein Cell.

[9] Rawat P., Mitra D. (2011). Cellular heat shock factor 1 positively regulates human immunodeficiency virus-1 gene expression and replication by two distinct pathways. Nucleic Acids Res..

[10] Pan X.Y., Zhao W., Wang C.Y., Lin J., Zeng X.Y., Ren R.X., Wang K., Xun T.R., Shai Y., Liu S.W. (2016). Heat Shock Protein 90 Facilitates Latent HIV Reactivation through Maintaining the Function of Positive Transcriptional Elongation Factor b (p-TEFb) under Proteasome Inhibition. J. Biol. Chem..

[11] Vicenzi E., Poli G. (1994). Ultraviolet irradiation and cytokines as regulators of HIV latency and expression. Chem. Biol. Interact..

[12] Iordanskiy S., Duyne R.V., Sampey G.C., Woodson C.M., Fry K., Saifuddin M., Guo J., Wu Y.T., Romerio F., Kashanchi F. (2015). Therapeutic doses of irradiation activate viral transcription and induce apoptosis in HIV-1 infected cells. Virology.

[13] Ortner V., Ludwig A., Riegel E., Dunzinger S., Czerny T. (2015). An artificial HSE promoter for efficient and selective detection of heat shock pathway activity. Cell Stress Chaperones.

[14] Ma X., Yang T., Luo Y., Wu L., Jiang Y., Song Z., Pan T., Liu B., Liu G., Liu J. (2019). TRIM28 promotes HIV-1 latency by SUMOylating CDK9 and inhibiting P-TEFb. eLife.

[15] Lin J., Zhang X., Lu W., Xu X., Pan X., Liang T., Duan S., Chen Y., Li L., Liu S. (2018). PR-957, a selective immunoproteasome inhibitor, reactivates latent HIV-1 through p-TEFb activation mediated by HSF-1. Biochem. Pharmacol..

[16] Zeng X., Pan X., Xu X., Lin J., Que F., Tian Y., Li L., Liu S. (2017). Resveratrol Reactivates Latent HIV through Increasing Histone Acetylation and Activating Heat Shock Factor 1. J. Agric. Food Chem..

[17] Jordan A., Bisgrove D., Verdin E. (2003). HIV reproducibly establishes a latent infection after acute infection of T cells in vitro. EMBO J..

[18] Ma X., Chen T., Peng Z., Wang Z., Liu J., Yang T., Wu L., Liu G., Zhou M., Tong M. (2021). Histone chaperone CAF-1 promotes HIV-1 latency by leading the formation of phase-separated suppressive nuclear bodies. EMBO J..

[19] Timmons A., Fray E., Kumar M., Wu F., Dai W., Bullen C.K., Kim P., Hetzel C., Yang C., Beg S. (2020). HSF1 inhibition attenuates HIV-1 latency reversal mediated by several candidate LRAs In Vitro and Ex Vivo. Proc. Natl. Acad. Sci. USA.

[20] Platt E.J., Wehrly K., Kuhmann S.E., Chesebro B., Kabat D. (1998). Effects of CCR5 and CD4 Cell Surface Concentrations on Infections by Macrophagetropic Isolates of Human Immunodeficiency Virus Type 1. J. Virol..

[21] Alemasova E.E., Lavrik O.I. (2019). Poly(ADP-ribosyl)ation by PARP1: Reaction mechanism and regulatory proteins. Nucleic Acids Res..

[22] Fossati S., Formentini L., Wang Z.Q., Moroni F., Chiarugi A. (2006). Poly(ADP-ribosyl)ation regulates heat shock factor-1 activity and the heat shock response in murine fibroblasts. Biochem. Cell Biol..

[23] Fujimoto M., Takii R., Katiyar A., Srivastava P., Nakai A. (2018). Poly(ADP-Ribose) Polymerase 1 Promotes the Human Heat Shock Response by Facilitating Heat Shock Transcription Factor 1 Binding to DNA. Mol. Cell Biol..

[24] Fujimoto M., Takii R., Takaki E., Katiyar A., Nakato R., Shirahige K., Nakai A. (2017). The HSF1-PARP13-PARP1 complex facilitates DNA repair and promotes mammary tumorigenesis. Nat. Commun..

[25] Yu D., Liu R., Yang G., Zhou Q. (2018). The PARP1-Siah1 Axis Controls HIV-1 Transcription and Expression of Siah1 Substrates. Cell Rep..

[26] Li Z., Wu J., Chavez L., Hoh R., Deeks S.G., Pillai S.K., Zhou Q. (2019). Reiterative Enrichment and Authentication of CRISPRi Targets (REACT) identifies the proteasome as a key contributor to HIV-1 latency. PLoS Pathog..

[27] Pang W., Zhang G., Jiang J., Zheng H., Zhang L., Zhang X., Song J., Zhang M., Zhu J., Lei A. (2017). HIV-1 can infect northern pig-tailed macaques (*Macaca leonina*) and form viral reservoirs in vivo. Sci. Bull..

[28] Archin N.M., Bateson R., Tripathy M.K., Crooks A.M., Yang K.H., Dahl N.P., Kearney M.F., Anderson E.M., Coffin J.M., Strain M.C. (2014). HIV-1 expression within resting CD4^+^ T cells after multiple doses of vorinostat. J. Infect. Dis..

[29] Ke R., Lewin S.R., Elliott J.H., Perelson A.S. (2015). Modeling the Effects of Vorinostat In Vivo Reveals both Transient and Delayed HIV Transcriptional Activation and Minimal Killing of Latently Infected Cells. PLoS Pathog..

[30] Lindquist S. (1986). The heat-shock response. Annu. Rev. Biochem..

[31] Cheng X., Belshan M., Ratner L. (2008). Hsp40 facilitates nuclear import of the human immunodeficiency virus type 2 Vpx-mediated preintegration complex. J. Virol..

[32] Low J.S., Fassati A. (2014). Hsp90: A chaperone for HIV-1. Parasitology.

[33] Wyzewski Z., Gregorczyk K.P., Szczepanowska J., Szulc-Dabrowska L. (2018). Functional role of Hsp60 as a positive regulator of human viral infection progression. Acta Virol..

[34] Nekongo E.E., Ponomarenko A.I., Dewal M.B., Butty V.L., Browne E.P., Shoulders M.D. (2020). HSF1 Activation Can Restrict HIV Replication. ACS Infect. Dis..

[35] Peng W., Hong Z., Chen X., Gao H., Dai Z., Zhao J., Liu W., Li D., Deng K. (2020). Thiostrepton Reactivates Latent HIV-1 through the p-TEFb and NF-kappaB Pathways Mediated by Heat Shock Response. Antimicrob. Agents Chemother..

[36] Bonner J.J., Chen D., Storey K., Tushan M., Lea K. (2000). Structural analysis of yeast HSF by site-specific crosslinking. J. Mol. Biol..

[37] Jaeger A.M., Pemble C.W.T., Sistonen L., Thiele D.J. (2016). Structures of HSF2 reveal mechanisms for differential regulation of human heat-shock factors. Nat. Struct. Mol. Biol..

[38] Nakai A., Tanabe M., Kawazoe Y., Inazawa J., Morimoto R.I., Nagata K. (1997). HSF4, a new member of the human heat shock factor family which lacks properties of a transcriptional activator. Mol. Cell Biol..

[39] Rabindran S.K., Giorgi G., Clos J., Wu C. (1991). Molecular cloning and expression of a human heat shock factor, HSF1. Proc. Natl. Acad. Sci. USA.

[40] Kroeger P.E., Sarge K.D., Morimoto R.I. (1993). Mouse heat shock transcription factors 1 and 2 prefer a trimeric binding site but interact differently with the HSP70 heat shock element. Mol. Cell Biol..

[41] Yamamoto N., Takemori Y., Sakurai M., Sugiyama K., Sakurai H. (2009). Differential recognition of heat shock elements by members of the heat shock transcription factor family. FEBS J..

