# Supplementary material for: PARP1 Might Substitute HSF1 to Reactivate Latent HIV-1 by Binding to Heat Shock Element
Source: Cells. 2022 Jul 29;11(15):2331. doi: 10.3390/cells11152331 (PMC9367301; doi:10.3390/cells11152331)
Supplement: Supplementary file 1 [file cells-11-02331-s001.zip › cells-1641032-supplementary.pdf]

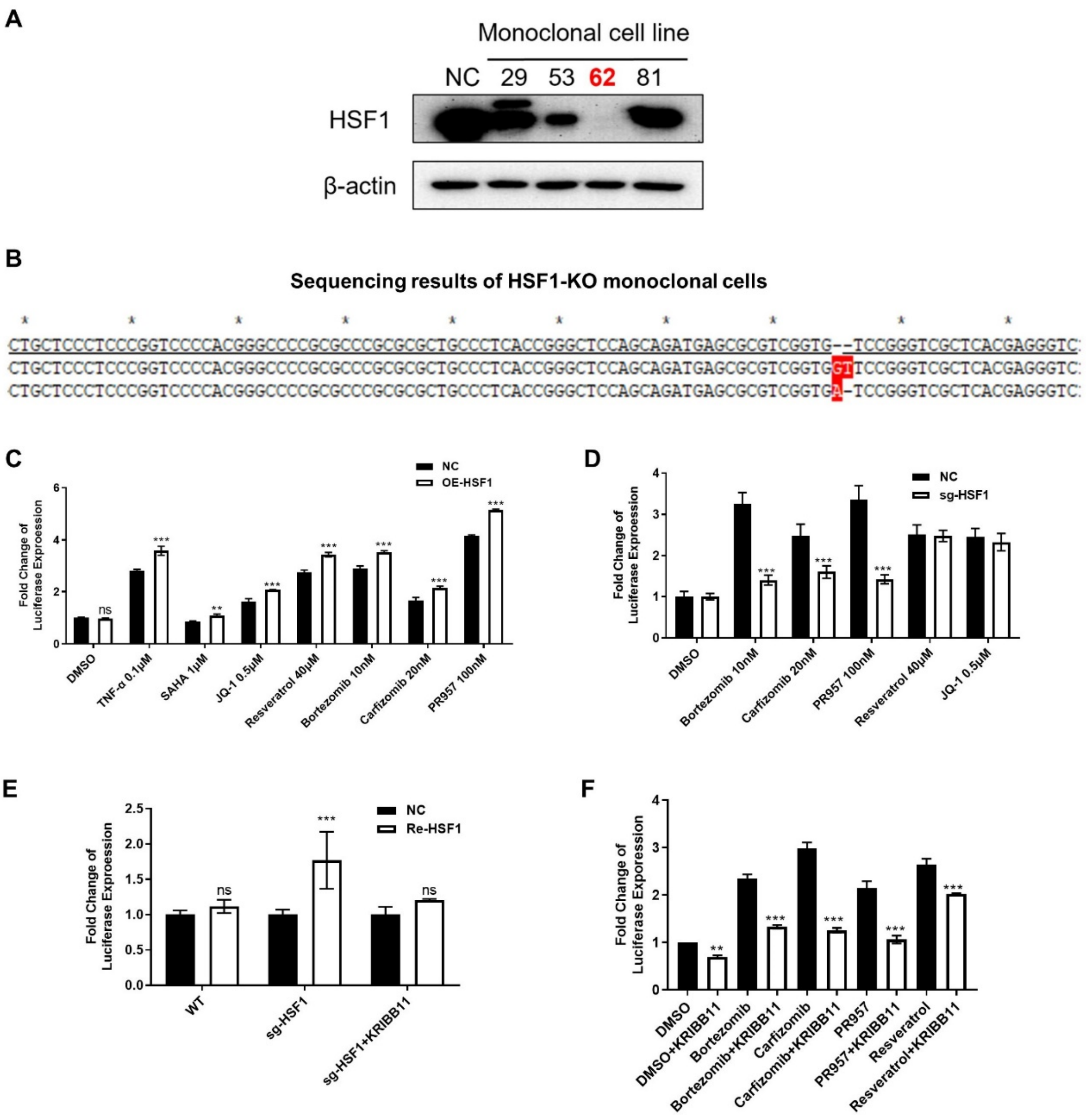

Figure S1. The depletion of HSF1 inhibited the HIV-1 promoter activity. **(A, B)** The knockout efficiencies of HSF1-KO monoclonal cells were evaluated by both western blot **(A)** and Sanger sequencing **(B)**. The Sanger sequencing results were derived from Clone No. 62. Two frameshift mutations which were "GT" and "A" bases insertion were detected in alleles. **(C)** TZM-bl cells were overexpressed with HSF1 proteins. Different LRAs were utilized to co-treat these cells. The fold changes of luciferase expression were evaluated 48 hours post treatment. **(D)** The endogenous HSF1 proteins were knocked out by sgRNA targeting HSF1 genes. Both wildtype and HSF1-KO cells were co-treated with different LRAs. The fold changes of luciferase expression were monitored 48 hours post treatment. **(E)** The HSF1-KO cells were re-expressed with HSF1-expressing plasmids. These cells were also treated with or without KRIBB11. The fold changes of luciferase within each group were quantitated and normalized to WT groups. **(F)** Wildtype TZM-bl cells were treated with different LRAs and / or KRIBB11. The fold changes of luciferase were evaluated as in **(E)**. All data were reported from three independent experiments as the mean± SEM and the p-value was defined as *$p < 0.05$, **$p < 0.01$ and ***$p < 0.001$ vs. control and ns was no statistical.

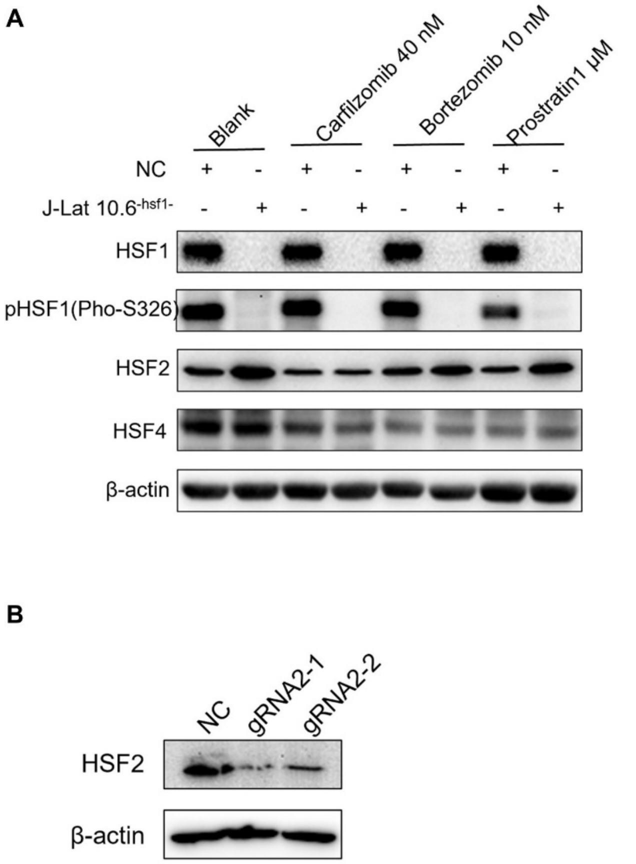

Figure S2. The expression of HSF2 was increased upon HSF1 knockout. **(A)** The expression of HSF family proteins in WT and HSF1-KO J-Lat 10.6 cells was evaluated by western blot. Different LRAs including carfilzomib (40 nM), bortezomib (10 nM), and Prostratin (1 μM) were used to co-treat these cells. **(B)** The knockout efficiencies of sgHSF2 in J-Lat 10.6 cells were evaluated by western blot.
